# Depression-Like Responses Induced by Daytime Light Deficiency in the Diurnal Grass Rat (*Arvicanthis niloticus*)

**DOI:** 10.1371/journal.pone.0057115

**Published:** 2013-02-20

**Authors:** Greg Leach, Widya Adidharma, Lily Yan

**Affiliations:** 1 Department of Psychology, Michigan State University, East Lansing, Michigan, United States of America; 2 Neuroscience Program. Michigan State University, East Lansing, Michigan, United States of America; Vanderbilt University, United States of America

## Abstract

Seasonal Affective Disorder (SAD) is one of the most common mood disorders with depressive symptoms recurring in winter when there is less sunlight. The fact that light is the most salient factor entraining circadian rhythms leads to the phase-shifting hypothesis, which suggests that the depressive episodes of SAD are caused by misalignments between the circadian rhythms and the habitual sleep times. However, how changes in environmental lighting conditions lead to the fluctuations in mood is largely unknown. The objective of this study is to develop an animal model for some of the features/symptoms of SAD using the diurnal grass rats *Arvichantis niloticus* and to explore the neural mechanisms underlying the light associated mood changes. Animals were housed in either a 12∶12 hr bright light∶dark (1000lux, BLD) or dim light∶dark (50lux, DLD) condition. The depression-like behaviors were assessed by sweet-taste Saccharin solution preference (SSP) and forced swimming test (FST). Animals in the DLD group showed higher levels of depression-like behaviors compared to those in BLD. The anxiety-like behaviors were assessed in open field and light/dark box test, however no significant differences were observed between the two groups. The involvement of the circadian system on depression-like behaviors was investigated as well. Analysis of locomotor activity revealed no major differences in daily rhythms that could possibly contribute to the depression-like behaviors. To explore the neural substrates associated with the depression-like behaviors, the brain tissues from these animals were analyzed using immunocytochemistry. Attenuated indices of 5-HT signaling were observed in DLD compared to the BLD group. The results lay the groundwork for establishing a novel animal model and a novel experimental paradigm for SAD. The results also provide insights into the neural mechanisms underlying light-dependent mood changes.

## Introduction

Seasonal Affective Disorder (SAD) is a recurrent major depressive disorder, in which episodes of depression occur in fall and winter followed by full remissions in spring and summer [1]. Light therapy is one of the most effective treatments for SAD, further supporting the association between mood and light [1], [2]. The mechanisms through which light affects mood are not well understood [3]. The current theories on SAD and light therapy have been focusing on the effects of light on circadian rhythms [2], [4], [5].

The objectives of the present study are to develop an animal model for some of the features/symptoms of SAD using the diurnal grass rats *Arvicanthis niloticus* and to explore the potential underlying neural substrates mediating the light-dependent mood changes. There are substantial differences in the circadian rhythms and in the direct light responses between nocturnal and diurnal species [6], [7]. Given the role that light and circadian rhythms play in SAD, a diurnal animal model has advantages over its nocturnal counterparts in elucidating the neurological responses that could be occurring in diurnal humans [8], [9], [10]. Furthermore, the grass rat shows depression-like behaviors when housed in short photoperiods, suggesting its potential use as an animal model for SAD [8], [11], [12].

Most previous works on animal models of SAD focus on the effects of photoperiod or day-length [11], [12], [13], [14], [15]. However, the daily ambient lighting conditions change over the seasons not only in day-length, but also in intensity and spectrum. Moreover, due to the use of artificial lights, the duration of daily light exposure that modern humans experience over the seasons does not fluctuate as much as the quality/intensity of the light [16], [17]. Hebert et al (1998) reported that the mean daily duration of time awake was similar in summer and winter (14.6 h vs. 14.9 h) [15]. In summer, the sunlight is brighter than in the winter, and in summer we also spend more time outdoors where the light is brighter than indoors. For example, the average light intensity is 800 lux in a bright office, whereas the intensity of sunlight is above 25,000 lux on a bright cloudy day and 100,000 lux on a bright sunny day [16]. Therefore, compared to day-length, light intensity could be a more salient determinant for regulating mood in humans. In the present study, animals were housed in 12∶12 hr light/dark cycle with either bright (BLD) or dim light (DLD) during the day, followed by assessment of depression- and anxiety-like behaviors. To evaluate the responses of the circadian system to changes in light intensity, daily rhythms in locomotor activity were analyzed in animals housed in BLD or DLD, respectively. In addition to the circadian mechanisms, the monoaminergic system has also been implicated in the etiology of SAD [18], [19], [20]. To explore the neural substrates underlying the behavioral changes, the serotoninergic signals were examined in the dorsal raphe nucleus (DRN) and several other brain regions. The results bring insights into the involvement of the circadian system and serotoninergic pathways that ultimately contribute to the light-dependent changes in mood.

## Experimental Procedures

### Animals

The grass rats (*Arvicanthis niloticus*) were obtained from a laboratory colony established with animals imported from sub-Saharan Africa by Dr. Laura Smale. The animals are equatorial, living in 12∶12 h light/dark cycle (LD) in their natural habitat. The laboratory colony was established in 1993 and has been maintained for almost two decades using outbred breeding. The animals were kept in laboratory under a 12∶12 h LD cycle with food (Prolab 2000 #5P06, PMI Nutrition LLC, MO) and water available *ad libitum*. The time of lights-on was defined as Zeitgeber time (ZT) 0. Light in the colony room was supplied by cool white fluorescent lights mounted on the ceiling. The light intensity at the center of the room was around 300 lux. The cages were placed on metal racks with multiple levels. The light intensity at the cage level ranged from 50 to 1000 lux depending on the location of the cage and the rack.

### Ethics

All experiments were carried out according to the National Institute of Health Guidelines for the Care and Use of Laboratory Animals. All experimental procedures were approved by the Michigan State University Animal Use and Care Committee (AUF-03-11-057).

### Experiment 1: Effects of daytime light intensity on depression-like behaviors

Two cohorts of adult male grass rats (4–6 months old) were used in this experiment. Adult males were used to be comparable with existing literatures [11], [12], [13], [14]. Paired littermates housed in same cages were assigned to two groups (n = 8/group in cohort 1, n = 5 or 8/group in cohort 2) under LD cycles with different daytime light intensity, i.e. bright light∶dark (BLD, 1000lux/1lux) and dim light∶dark (DLD, 50lux/1lux). Following the assignment into the two groups, animals were singly housed in plexiglass cages (47×25×20 cm for cohort 1; 38×33×16.5 cm for cohort 2) placed in cabinets with controlled light intensity. A PVC pipe (7.5 cm diameter, 12 cm long) was provided in the cage for enrichment and as a hut for the animal. Light was supplied by cool white fluorescent lights and the intensity was determined by averaging the measurements from the top and four sides of the cage. The cages for the BLD group were located about 10 cm below the light source, while the cages for the DLD group were at least 1.5 meters away from the light source with shades over the top of the cages to block the direct light exposure. After 4 weeks, the following behavioral tests were performed to assess the depression-like behaviors. There was at least a 3 to 5 day interval between each test. After the tests, animals were left undisturbed for another 5 days before being sacrificed for brain analysis.

#### Saccharin solution preference (SSP) test

Sweet-taste solution preferences are commonly used to detect anhedonia in animal models of depression [21], [22]. In addition to the regular supply of water and food, grass rats were supplied with a bottle of 1.0% saccharin (Sigma, MI) dissolved in tap water over 3 days for cohort 1 and 4 days for cohort 2. The saccharin concentration was selected based on previous experiments with diurnal sand rats and diurnal grass rats [11], [12], [13], [23]. The amount of saccharin solution and water intake was measured every 24 h. Saccharin solution preference (SSP) was calculated daily as a ratio of saccharin solution consumption out of total liquid (saccharin solution+water) consumption. The first 24 hr of the exposure was used as a training session for the animals to associate the location of the bottle with the taste, and the SSP over this period was defined as the baseline. There was no difference in the baseline preference between the two groups (BLD: 27±6%; DLD: 24±7%; t-test, p = 0.81). If the sweet taste is a hedonic cue, an increase in the SSP in the following days is expected. Therefore, changes in the SSP for each of the subsequent testing days over the baseline were calculated for each animal and analyzed statistically. One animal in cohort 1 DLD group was identified as an outlier (2 standard deviations away from group mean) and removed from the analysis.

#### Forced swim test (FST)

This test was performed as described in previous studies [11], [12], [23], [24]. The test involved two exposures, a pre-test and a test, to a cylindrical pool (35.5 cm tall×30.5 cm diameter) filled with 25 cm of water maintained at 28–30°C. Water in the tank was changed after each animal. For the pre-test, each grass rat was placed in the water tank for 10 min. Twenty-four hours later, the animal was placed in the water again and videotaped for a 5 min test session. The time that animals were immobile was scored from the videotapes by observers who were unaware of the experimental conditions of each animal. All the tests were performed during the daytime between ZT6 and ZT10 with the order alternated between individuals in the BLD and DLD groups to control the time of testing. The light intensity in the testing room was around 300 lux. The immobility was scored when the grass rats were passively floating without movements other than those necessary for keeping the head above water. Climbing was scored when the animals showed upward movements with their forepaws touching the wall of the water tank. Swimming was defined as horizontal movements other than passive floating [24]. One animal in cohort 1 DLD group and one in cohort 2 BLD were identified as outliers (2 standard deviations away from group mean). Another animal in cohort 2 BLD group dived to the bottom during the training session and stayed immobile for almost the entire 5-minute testing session. Those 3 animals (out of the total 29) were excluded from the analysis for FST.

### Experiment 2: Effects of daytime light intensity on anxiety-like behaviors

Adult male grass rats (4–6 months old) were singly housed in plexiglass cages (38×33×16.5 cm) under BLD or DLD conditions as described in experiment 1 (n = 7/condition). After 4 weeks, the following behavioral tests were performed to assess the anxiety-like behaviors. The tests were performed during the daytime between ZT4 to ZT6 in a testing room with light intensity around 300 lux at the center of the room. There was at least a 3 to 5 day interval between the tests.

#### Open field test (OFT)

A white plexiglass box (80 cm length×80 cm width ×60 cm height) was used. The floor of the box was outlined by 4 lines evenly spaced in each direction, which resulted in 25 squares (5 squares long and 5 squares wide). The squares were used to indicate the location of the animals during the test: 16 squares were aligned with the edge of the walls (outer squares), and 9 squares were inside of the outer squares (center squares). The grass rats were put in the corner square with their heads facing the center and were allowed to explore freely for 5 min. The behaviors were videotaped and scored manually for the time spent or the incidence of line crossing within each region. The area was wiped clean with a 70% alcohol solution between the tests for each animal.

#### Light/dark box test

The light/dark box consisted of two compartments, a dark (30 cm length×80 cm width×60 cm height) and a light (50 cm length×80 cm width×60 cm height) area connected by an opening (15 cm height×10 cm width) between the two. The box was constructed of plastic. The light intensity in the dark side was less than 5 lux. The light region was not covered on the top and received room light. The small opening connecting the two chambers allowed the animals to freely enter either area. Animals were placed in the light side of the chamber facing the opening to the dark chamber and allowed to move freely between the two compartments for a 5 min session. Behaviors were videotaped and scored for time and frequency of visits to the dark part of the box. An animal was considered to be in one of the compartments when its head and the two front paws were in that area of the box. At the end of the session animals were returned to their home cages and the area was wiped clean with a 70% alcohol solution.

### Experiment 3: Effects of daytime light intensity on daily rhythms in locomotor activity

Adult male grass rats (4–6 months old) were singly housed in plexiglass cages (38×33×16.5 cm) under BLD or DLD conditions as described in experiment 1 (n = 6/condition). In-cage locomotor activity was recorded by a motion sensor mounted on top of each cage. General locomotor activities were recorded in 5-minute bins using VitalView (Minimitter, Inc.) for four weeks. The following circadian parameters were examined based on the data collected in the last 14 days: daily activity, day/night activity ratio, the duration of the active phase, time and stability of activity onset and offset. Daily activity was calculated by averaging the total amount of activity per day. Day/night activity ratio was calculated as the average ratio between the activity during the 12 hr light and 12 hr dark phase over 14 days for each animal. The duration of active phase was calculated based on the daily activity onset and offset time by creating actograms using the ClockLab (Actimetrics, Inc.). Entrainment stability was calculated as the standard deviation of the daily activity onset or offset time over 14 days.

### Experiment 4: Effects of daytime light intensity on serotonin (5-HT) immunoreactivity (ir)

At the end of experiment 1, animals (n = 6/group from cohort 1, randomly picked) were euthanized (pentobarbital, 200 mg/kg, ip) between ZT5 to ZT6 and perfused transcardially using 50 ml saline followed by 100 ml 4% paraformaldehyde in 0.1 M phosphate buffer. After the perfusion, the brains were post-fixed, cryoprotected, and sectioned at 40 µm using a cryostat (Leica, IL).

#### Immunocytochemistry (ICC)

ICC for serotonin (5-HT) was carried out on every third section using methods as described in previous studies [25], [26]. The sections were first incubated with an antibody against 5-HT (1∶10,000, NT-102 5HTrab, made in rabbit, Protos Biotech, NY) at 4°C for 3 days, then rinsed in 0.1 M phosphate buffer before being incubated in secondary antibody (biotinylated Goat anti-rabbit, 1∶1000, Vector lab, CA) at 4°C overnight. Sections were then processed with the avidin-biotin-immunoperoxidase technique using the VECTASTAIN Elite ABC system (Vector lab) following the protocol provided by the manufacturer. Finally, using 3′3′-diaminobenzidine (DAB, 2%) as the chromogen, the 5-HT-containing cell bodies and fibers were stained brown. Following the ICC reaction, sections were mounted on slides, dehydrated with alcohol rinses, cleared with xylene, and coverslipped with Permount (Fisher Scientific, NJ, USA).

#### Quantitative analysis of ICC results

For quantification, images of sections stained with 5-HT were captured using a CCD video camera (CX9000, MBF bioscience, VM, USA) attached to a light microscope (Nikon Instruments Inc., NY, USA). The camera and microscope setting was identical for each image. All the images were analyzed by experimenters who were unaware of the experimental conditions of the animals. 5-HT-ir was analyzed in the following midbrain and forebrain regions: the dorsal raphe nucleus (DRN), periaqueductal gray (PAG), medial cingulate cortex (Cgc), and the suprachiasmatic nucleus (SCN). Sections containing the areas of interest (4–8 sections/area) corresponded to the rostro-caudal extent from plane 19 to 24 (Bregma −0.3 to −1.4 mm) for Cgc, plane 22 to 24 (Bregma −0.92 to −1.4 mm) for SCN, plane 43 to 46 (Bregma −6.3 to −7.04 mm) for PAG and plane 47 to 52 (Bregma −7.3 to −8.32 mm) for DRN of a rat brain atlas [27]. In the DRN, sections were analyzed across the rostro-caudal extent from level 1 to level 4 [28]. The sections from levels 1 to 3 where the 5-HT neurons are clustered at the center were grouped as rostral, while those from level 4 where the clusters of the 5-HT neurons are lateralized were defined as middle DRN. The caudal DRN contains fewer 5-HT cells when compared to the rostral and middle portions [29] and therefore was not analyzed in the present study. The sections were analyzed by cell counting or density measurement as previously described [30]. The density of fibers/terminals was quantified using NIH Image J. The size of each area of interest being measured was kept consistent across the sections/animals. A threshold that distinguished the staining from the background was also set consistently for each area. The fraction of pixels above the threshold in the area of interest was measured and averaged across the sections from the same region. The average fraction represented the density of staining per animal.

#### Statistical Analysis

For experiment 1, the SSP data were analyzed by a mixed factor ANOVA with light conditions as a main factor and day as a Repeated Measure factor; the FST data were analyzed with unpaired two-tailed t-test. For all the comparisons in experiment 1, the differences were considered significant when p<0.05. For experiment 2, the effect of light intensity on each measurement from the two groups was analyzed with un-paired two-tailed t-test. For experiment 3, the daily activity profile was analyzed by a two-way ANOVA (light conditions X time of the day). The difference was considered significant when p<0.05. The effect of lighting condition on the 7 measures for rhythmic behaviors i.e. daily activity, day/night activity ratio, the duration of the active phase, time and stability of activity onset and offset was analyzed using unpaired two-tailed t-test, followed by a Bonferroni correction for multiple comparisons. The differences were considered significant when p<0.007 (Bonferroni correction: 0.05 divided by the number of comparisons). For experiment 4, the effect of light intensity on the immunostaining of 5-HT in each brain region was analyzed using un-paired two-tailed t-test. In all cases, differences were considered significant when p<0.05.

## Results

### Dim light during the day induces depression-like behavioral responses

**Figure 1:**
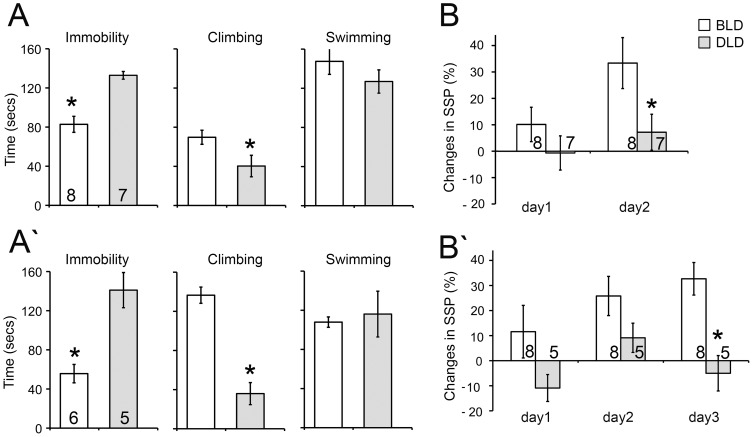
Effects of light intensity on depression-like behavioral responses. Bar graphs on the left side show the duration for immobility, climbing and swimming during FST on the test day for cohort 1 (A) and cohort 2 (A′). The changes in preference for SSP are shown over two days for cohort 1 (B) and over 3 days for cohort 2 (B′). Results are displayed as mean ± SEM. Numbers on each column indicate the sample size. * indicates p<0.05.

Effects of light intensity on SSP and on immobility/climbing behaviors during FST were observed in two separate cohorts of animals (Fig. 1). For cohort 1, DLD animals showed a significant increase in immobility (t-test, p<0.01) and a significant decrease in climbing (t-test, p<0.05) compared to those in BLD during FST (Fig. 1A). There was no difference in swimming between the BLD and DLD animals (Fig. 1A, t-test, p = 0.3). During the test of SSP, the baseline SSP was comparable between the two groups (BLD: 27±6%; DLD: 24±7%; t-test, p = 0.81). Over the days of exposure, a marginally significant effect of lighting condition and a significant effect of day emerged (Fig. 1B, mixed factor ANOVA, effect of light: F_1,13_ = 4.12, p = 0.06; effect of day: F_1,13_ = 11.33, p = 0.005; interaction: F_1,13_ = 2.78; p = 0.12).

To further confirm the behavioral effects, a 2^nd^ cohort of animals was subjected to the same lighting paradigm and tested the same way as those in the 1^st^ cohort. The 2^nd^ cohort of animals behaved very similarly to those in the 1^st^ cohort (Fig. 1A′, B′). During FST (Fig. 1A′), the DLD group showed a significant increase in immobility (t-test, p<0.01) and a decrease in climbing (t-test, p<0.01) compared to the BLD group, without any difference in swimming (t-test, p = 0.7). There was no difference in the baseline SSP between the two groups (BLD: 38±8%; DLD: 32±12%; t-test, p = 0.7). A significant effect of lighting condition was observed in the changes in SSP (Fig. 1B′, mixed factor ANOVA, effect of light: F_1,11_ = 6.16, p = 0.03; effect of day: F_2,11_ = 4.17, p = 0.03; interaction: F_2,11_ = 2.57, p = 0.10).

### Effects of daytime light intensity on anxiety-like behaviors

**Figure 2:**
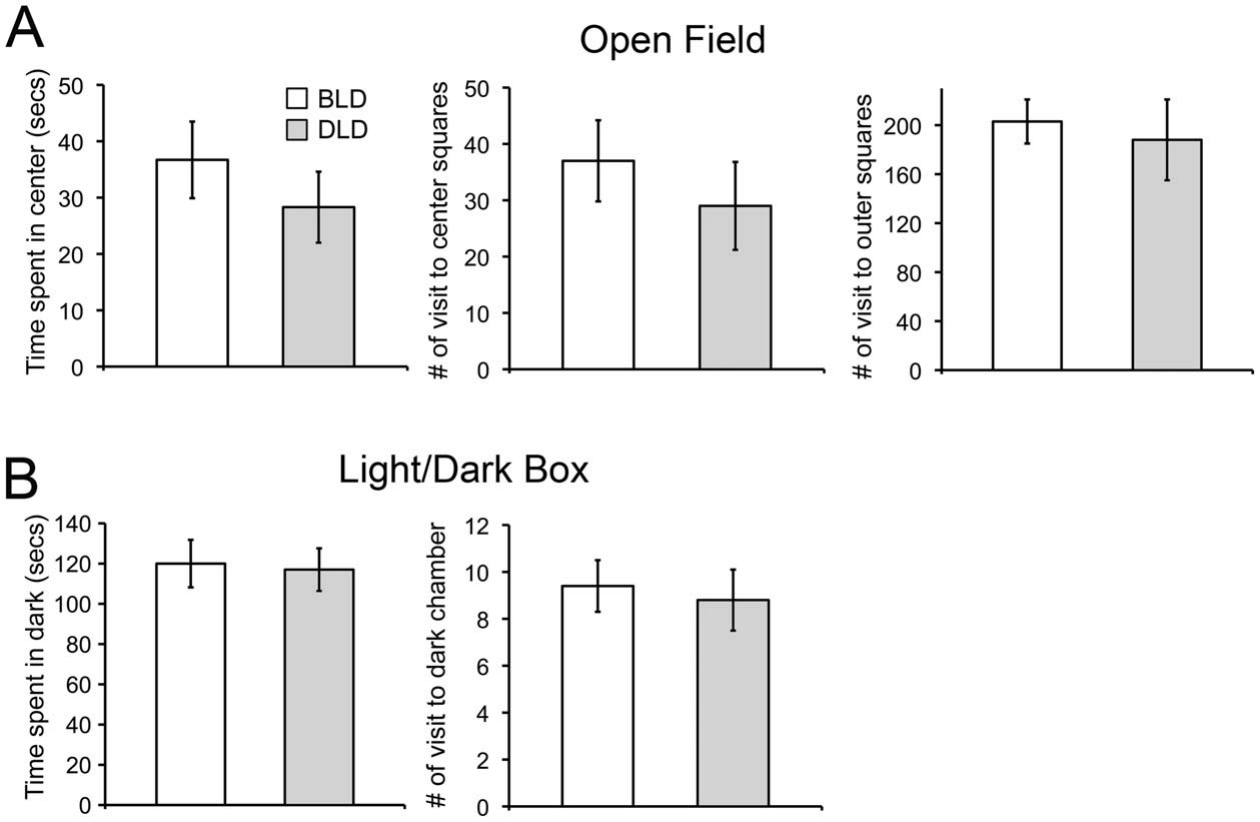
Effects of daytime light intensity on anxiety-like behaviors assessed by open field (A) and light/dark box test (B). The results are presented as mean±sem (n = 7).

During the open field test (OFT), both BLD and DLD groups of animals spent most (∼90%) of the time running through the outer squares or the edge of the arena. There was no significant difference in the time spent in the center squares or the incidence of entering into a center square (Fig. 2A, left panel, t-test, p>0.05). The incidence of the animals entering an outer square was not different either (Fig. 2A, right panel, t-test, p>0.05).

During the light/dark box test, the animals spent more time in the light than in the dark chamber (181±7 vs. 118±7 sec), which reflects their diurnal nature. There was no significant difference in the light/dark preference between the two groups measured by the time spent in or the number of visits to the dark chamber (Fig. 2B, t-test, p>0.05).

### Effects of daytime light intensity on daily rhythms

**Figure 3:**
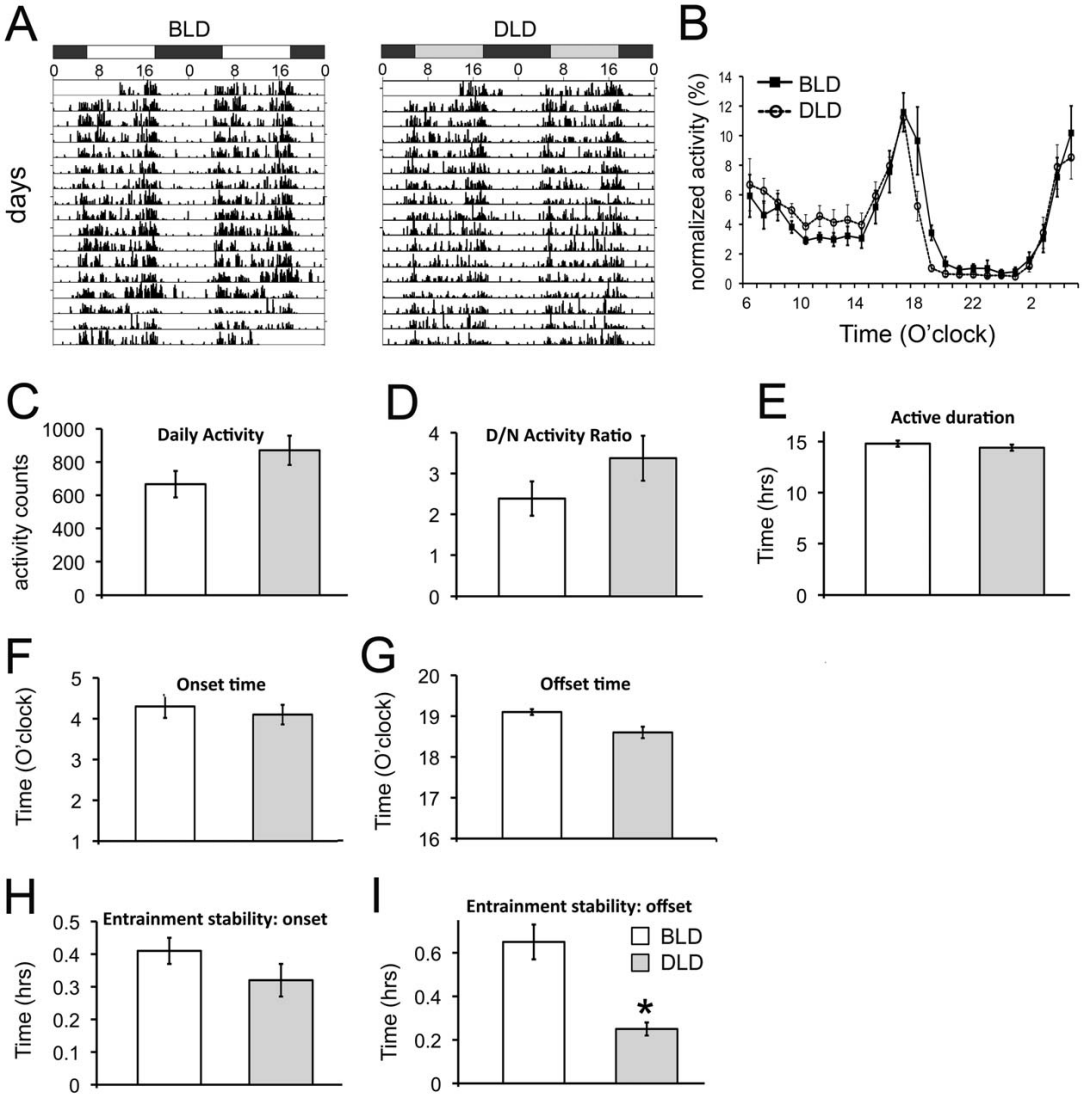
Effects of light intensity on circadian rhythms. The actograms (A) depict representative daily activity from one animal for each condition over two weeks. The line graph (B) shows the average activity for each condition over the course of a day. The daily activity was averaged over the last 14 days of recording, and normalized by dividing by the total daily activity and is displayed as a percentage of total activity for each individual animal. The normalized activity was then averaged within each condition and shown in the line graph. The error bars represent the SEM between individuals within each condition. The bar graphs show the average values from each condition for each of the circadian parameters observed: daily activity (C), day/night activity ratio (D), duration of the active phase (E), activity onset (F) and offset (G) times, and entrainment stability of both onset (H) and offset (I). Results are displayed as mean ± SEM (n = 6). * indicates p<0.007 (with Bonferroni correction for multiple comparisons).

The effects of daytime light intensity on daily behavioral rhythms were minor (Fig. 3). A clear diurnal pattern of activity was observed in animals in both BLD and DLD (Fig. 3A). A quantitative analysis of the daily activity profile did not reveal any significant effect of lighting condition (Fig. 3B, two-way ANOVA, effect of light: F_1,240_ = 0, p = 1; effect of time: F_23,240_ = 22.1, p = 0; interaction: F_23,240_ = 1.11, p = 0.33). We also analyzed several parameters for daily rhythms (Fig. 3C–I). No significant effect of lighting condition was found in total daily activity (p>0.05), day/night activity ratio (p>0.05), active phase duration (p>0.05), activity onset time (p>0.05) and offset time (Fig. 3G, t-test, p = 0.008). A significant difference between the BLD and DLD animals was only found in their entrainment stability measured by offset time (Fig. 3I, t-test, p = 0.001), indicating that the variability of offset time within an individual animal across days is larger in animals in BLD compared to those in DLD. In other parameters examined, there was no significant difference between the BLD and DLD groups.

### Dim light during the day attenuates 5-HT-ir

**Figure 4:**
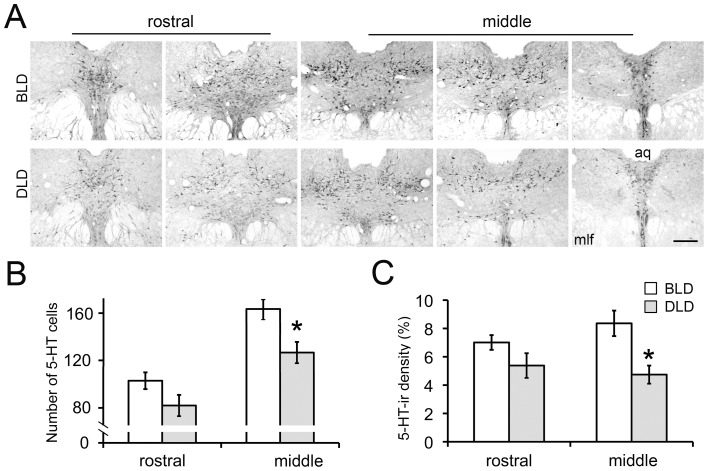
Effects of light intensity on 5-HT signals in the DRN. (A), representative photomicrographs of 5-HT staining in the DRN across the rostral-caudal extent in BLD and DLD group. Histograms show the number of 5-HT neurons (B) and the density of 5-HT-ir (C) in the rostral and middle portion of the DRN of animals from the BLD and DLD groups. Results are displayed as mean ± SEM (n = 6). * indicates p<0.05. Scale bar, 250 µm. aq, aqueduct; mlf, medial longitudinal fasciculus.

**Figure 5:**
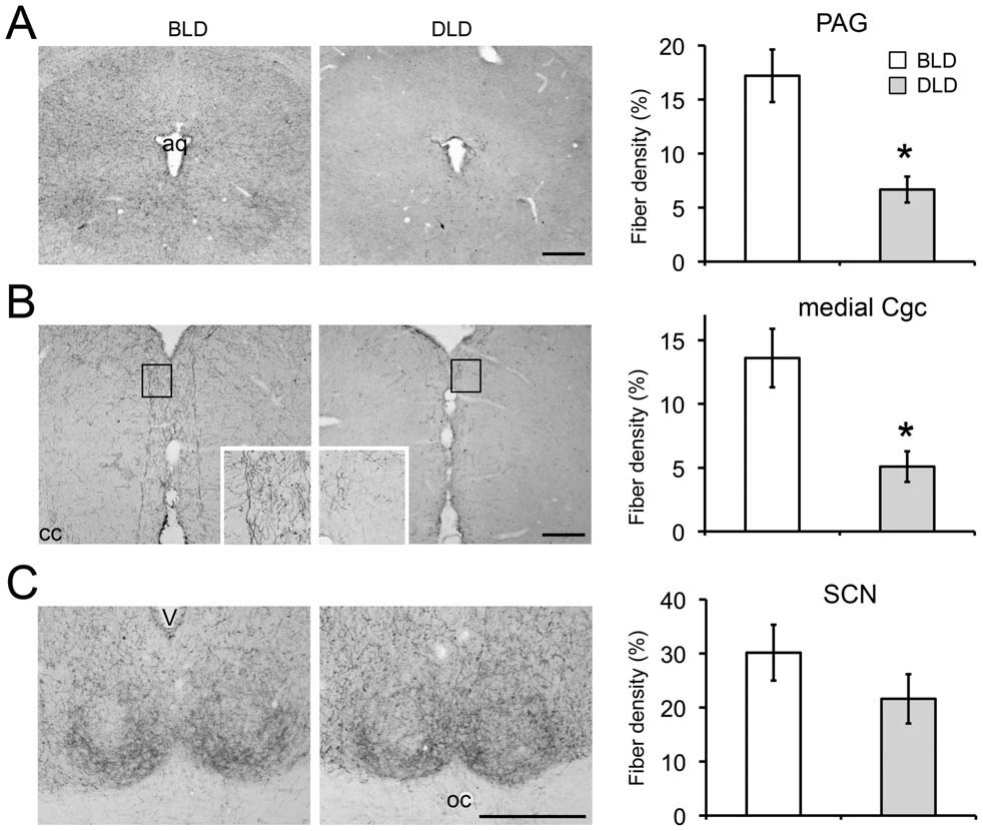
Effects of light intensity on 5-HT expression in midbrain and forebrain regions related to the regulation of mood or circadian rhythms. The histograms and representative photomicrographs depict 5-HT staining in each condition in the PAG (A), Cgc (B), and SCN (C). Each set of histograms show the density of 5-HT-ir fibers in each area. Results are displayed as mean ± SEM (n = 6). * indicates p<0.05. Scale bar, 250 µm. aq, aqueduct; CC, corpus callosum; OC, optic chiasm; V, third ventricle.

To identify the neural substrates underlying the light-dependent depression-like behaviors, 5-HT-ir was examined in the brains of the 1^st^ cohort of animals (Fig. 4, 5). In the dorsal raphe nucleus (DRN), the 5-HT staining was analyzed in both the rostral and middle portion of the nucleus (Fig. 4A). Two-way ANOVA revealed a significant effect of lighting condition and rostrocaudal region on the number of 5-HT-ir neurons (Fig. 4B, effect of light: F_1,20_ = 9.75, p = 0.005; effect of region: F_1,20_ = 9.52, p = 0.006; interaction: F_1,20_ = 0.05, p = 0.82). A significant effect of light was also found in the density of 5-HT-ir in the DRN (Fig. 4C, effect of light: F_1,20_ = 12.24, p = 0.002; effect of region: F_1,20_ = 0.22, p = 0.64; interaction: F_1,20_ = 1.75, p = 0.2). A planned t-test revealed that there was a significant difference in the number of 5-HT-ir cells and in the density of 5-HT-ir between the BLD and DLD group only in the middle portion (p<0.01) but not in the rostral portion (p = 0.1) of the DRN.

The 5-HT fiber staining was also quantified in midbrain and forebrain regions that are related to the regulation of mood or circadian rhythms (Fig. 5). A significant decrease of 5-HT fiber staining was observed in the periaqueductal gray (PAG, Fig. 5A, t-test, p<0.01) and in the medial cingulate cortex (Cgc, Fig. 5B, t-test, p<0.01). It is noteworthy that in the medial Cgc, there is a distinct pattern of net-like 5-HT fibers located in layer 1 of cerebral cortex in BLD animals but absent in the DLD animals (Fig. 5B). In the suprachiasmatic nucleus (SCN), the principal brain clock in the hypothalamus, dense 5-HTergic inputs were observed in both BLD and DLD groups without a significant difference (Fig. 5C, t-test, p = 0.25).

## Discussion

Using a diurnal rodent model, the present study demonstrates that decreasing daytime light intensity is effective in producing depression-like behaviors, but without apparent disruption in daily rhythms. The depression-like behaviors are associated with attenuated indices of 5-HTergic functions in brain regions that are involved in mood regulation.

The depression-like behaviors were assessed using the FST and SSP test (Fig. 1). Significant effects of light intensity on these behavioral measures were first observed in one cohort of animals. To our knowledge, the behavioral effects under this paradigm (bright vs. dim light) have not been reported previously. Thus, to ensure the results are reliable and replicable, a second cohort of animals from different breeding pairs was tested under the same paradigm. Consistent results were observed in the two separate cohorts of animals with the DLD group showing a higher level of depression-like behaviors compared to BLD group. The DLD groups showed longer immobility and less climbing compared to the BLD group. There was no difference in daily activity between the BLD and DLD groups (Fig. 3C), suggesting that light intensity within this range has no direct effects in inhibiting or promoting locomotor activities i.e. climbing or swimming behaviors during the FST. The animals in the two cohorts were from different breeding pairs, housed in cages of slightly different sizes and were tested at different times of the year with possible differences in the temperature and/or humidity in the facility. The protocol for SSP test was also slightly different; the duration of sweet solution exposure was 3 days for the first while it was 4 days for the second cohort. Even with these caveats, the overall pattern of the behavioral responses was consistent, indicating that the effects of light intensity on depression-like behaviors are quite robust. In addition, since the FST and the SSP assess different aspects of behavior/physiology [21], [24], the fact that the effect of light intensity was observed in both tests is very encouraging for the validity of this model. The effects of light intensity on anxiety-like behaviors were also assessed using open field and light/dark box (Fig. 2). No significant differences were found between the BLD and DLD groups in those tests, suggesting the effects of light intensity utilized in the present study were specific to depression-like behavioral responses.

Although the 1000 lux for the BLD group was brighter than the commonly used laboratory condition, it was still much dimmer compared to the outdoor lights that the animals experience in nature, which is above 25,000 lux even on a cloudy day [16]. It should also be noted that although the light intensity at the cage level was tightly controlled, the actual light intensity the animals experienced could vary depending on the physical location of the animals inside the cages. For example, under the feeding tray or water bottle was not as bright as the areas receiving direct light exposure. Moreover, each cage was equipped with a PVC pipe as enrichment that could be used as a shelter. The light intensity inside the PVC pipe was less than 10 lux. The animals do spend time inside the PVC pipe during the day, which may reflect their habit of living in underground burrows in nature. Therefore, the light intensity at the cage level reported was the maximum intensity, but the animals could voluntarily tune it down by choosing different locations inside the cages. It is also noteworthy that FST was performed in 300 lux, therefore, animals in the BLD condition were exposed to dimmer light during FST, while animals in the DLD condition were exposed to brighter light during FST, compared to their previous lighting conditions. Stress responses caused by changes in light intensity may affect FST performance. It is unknown whether the changes in light intensity, which differed in direction and magnitude between the BLD and DLD groups, elicited equivalent stress responses. In future studies, a group that is maintained in identical lighting conditions before and during FST should be examined. However, this does not change the main conclusion of the study, which suggests light intensity can influence depression-like behaviors in grass rats.

Current hypotheses about SAD focus on chronobiological mechanisms and suggest that the depression episodes stem from the dysregulation of circadian rhythms associated with light deficiency [2], [4], [5]. When the daily rhythms in general activity were compared between animals housed in BLD and DLD conditions, any differences detected were very subtle (Fig. 3). There was no difference in the amount of daily activity or day/night activity ratio between the two groups, suggesting that the depression-like responses in the DLD group were not due to lack of physical activity (Fig. 3C). The offset time for the DLD group was about 30 min earlier than the BLD group, but the difference was not statistically significant (Fig. 3G). Additionally, there was no difference in the activity onset time in the morning (Fig. 3F). Among the parameters for daily rhythms, the only one that was affected by light intensity was the entrainment stability measured by the offset time of activity (Fig. 3I). The DLD animals showed less variability in the offset time, which suggests the entrainment of the DLD group was more stable compared to the BLD group (Fig. 3I). Therefore, our data revealed no signs of disturbance or phase shifts in the daily rhythms of the DLD group. The results seem to suggest that in addition to the dysregulation of circadian rhythms, circadian-independent mechanisms are also likely involved in the light-dependent mood changes [31].

The monoamine hypothesis, which is one of the leading explanations for depression, has been implicated in SAD [3], [18]. Antidepressant drugs enhancing serotonin (5-HT) signaling are effective in alleviating the depression symptoms in SAD patients [19], [20], [32]. Meanwhile, reducing 5-HT synthesis through tryptophan depletion prevents the remission of depression symptoms in SAD patients following light therapy [33], [34]. The present study examined the 5-HT immunoreactivity (ir) in the brains and the results revealed a reduction in 5-HT-ir in the DRN, PAG and mCgc of animals that showed depression-like behaviors in the DLD group (Fig. 4, 5). The DRN is a heterogeneous midbrain structure containing 5-HT cells that have been implicated in depression [35]. The reduction of 5-HT-ir in the DRN further validates this model and supports the idea that the attenuation of 5-HT signaling underlies the depression symptoms in SAD [3]. The DRN can be divided into three parts along the rostrocadual axis [29]. The neurons in the rostral part mainly project to basal ganglia, while those in the middle part, especially in the dorsal and lateral region, send efferent projections to limbic and cortical regions that are involved in emotional behaviors (reviewed in [36]). By analyzing the 5-HT-ir along the rostrocaudal axis, the results revealed that the reduction of 5-HT-ir in the animals showing depressive behaviors was more confined in the middle but not in the rostral DRN (Fig. 4). This finding is consistent with the idea of functional heterogeneity of this nucleus, suggesting that the neurons in the middle portion of the DRN may play a more important role compared to those in the rostral portion in regulating emotional behaviors. The 5-HT fibers were also analyzed in a few brain regions receiving 5-HTergic innervations (Fig. 5). A significant decrease in 5-HT-ir in DLD compared to BLD group was observed in the PAG and the Cgc (p<0.01) but not in the SCN (p = 0.25), suggesting the changes in 5-HT signals are not uniform in the brain but rather region specific. Within those regions, the PAG and the medial prefrontal cortex (mPFC) have been implicated in mood regulation, with 5-HT as an important neuromodulator influencing the function of PAG and mPFC, among many other brain regions [35], [37], [38], [39]. The decreased level of the 5-HT measurements in the PAG and mPFC in DLD group suggests that the deficiency of 5-HT signals underlies the depression-like behavior provoked by daytime dim light.

An important question that needs to be answered is how lighting conditions affect 5-HTergic tone in the brains of diurnal grass rats. The DRN receives direct retinal projections in a few rodent species i.e. Mongolian gerbils, Chilean degus and lab rats [40], [41], [42], [43]. However, no direct retinal projections were observed in the DRN of mice [44]. Light information can also reach to the DRN through indirect pathways. One of the major inputs to the DRN is from orexin neurons [45], [46]. Recently, we found that in the diurnal grass rats, acute light exposure activates orexin neurons and neurons in the DRN; moreover, blocking the orexinergic signaling inhibits light-induced activation in the DRN [26]. Therefore, the orexinergic pathways could be one of the mechanisms mediating the effects of light on 5-HT and other monoaminergic systems. This hypothesis will be further tested in future studies.

The results from the present study demonstrated that decreased daytime light intensity, which occurs in nature during the transition from summer to fall/winter, induces depressive behaviors in diurnal grass rats. The depression-like behaviors are associated with attenuated indices of 5-HT tone, which further validates this model of SAD. Another important validation for an animal model of SAD is the predictive/treatment validity. Using the diurnal fat sand rats, Drs. Einat, Kronfeld-Schor and their colleagues have shown that the depression-like behavior induced in diurnal sand rats by short days could be alleviated by bright light therapy and by antidepressant treatment [47], [48], [49]. Lacking the predictive/treatment validity at this time is a major limitation of the diurnal grass rat model, which will be addressed as the next step in future studies.

In contrast to the generally accepted circadian hypothesis, the depressive responses induced by daytime dim light seem to be independent of any major changes in the circadian system. Lighting conditions change over the seasons with respect to intensity, duration and spectrum. Changes in each parameter could affect distinct neural pathways involving circadian-dependent and/or circadian-independent mechanisms [31], [50]. Using an adequate animal model combined with specific experimental paradigms, it will be possible to distinguish the impact of each parameter of the environmental lighting conditions on the affective responses as well as the underlying neural substrates. Such knowledge will contribute to the development of effective preventative and therapeutic strategies in light-associated mood disorders.
